# Supplementary material for: CFTR mRNAs with nonsense codons are degraded by the SMG6-mediated endonucleolytic decay pathway
Source: Nat Commun. 2022 Apr 29;13:2344. doi: 10.1038/s41467-022-29935-9 (PMC9054838; doi:10.1038/s41467-022-29935-9)
Supplement: Supplementary file 1 — Supplementary Information [file 41467_2022_29935_MOESM1_ESM.pdf]

Supplementary Information

CFTR mRNAs with nonsense codons are degraded by the SMG6-mediated endonucleolytic pathway

Edward J. Sanderlin[1], Melissa M. Keenan[1], Martin Mense[2], Alexey S. Revenko[1], Brett P. Monia[1], Shuling Guo[1], and Lulu Huang[1 *]

[1]Ionis Pharmaceuticals, Inc., Carlsbad, CA, USA, [2]Cystic Fibrosis Foundation Therapeutics Lab, Cystic Fibrosis Foundation, Lexington, MA, USA.

*Correspondence: Lulu Huang, Ionis Pharmaceuticals, Inc., 2855 Gazelle Ct, Carlsbad, CA 92010, USA

Email: lhuang@ionisph.com

1. Supplementary Figures 1-11……………………………………………………….Page 2-23
2. Supplementary Table 1……………………………………………………………. Page 24

Supplementary Figure 1

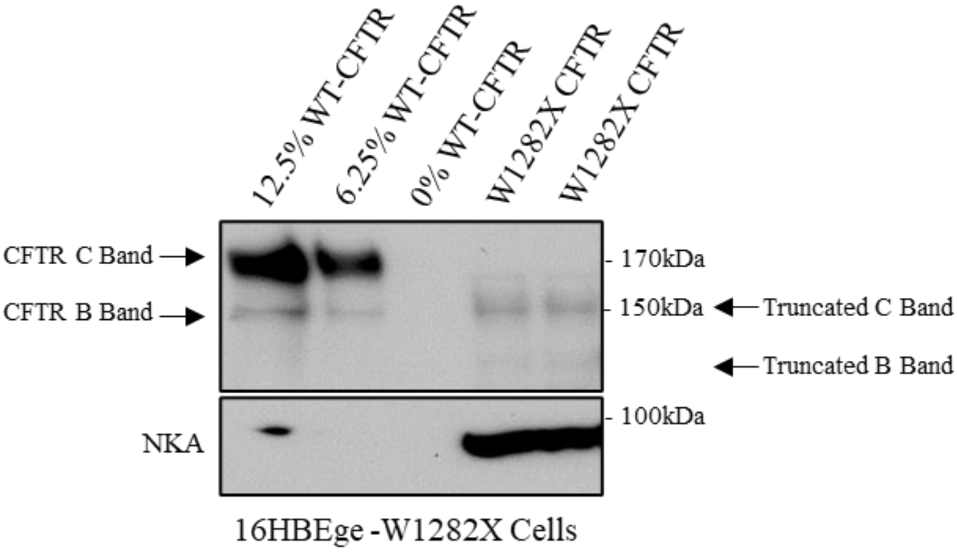

Supplementary Figure 1: CFF-16HBEge cells harboring the CFTR W1282X nonsense mutation have detectible C-truncated CFTR protein products. Qualitative western blot analysis of C-truncated CFTR W1282X proteins in CFF-16HBEge-W1282X cells. Na+/K+-ATPase (NKA) was used as a loading control. These data are representative of n=3 independent biological replicates. Source data are provided as a Source Data file.

Supplementary Figure 2

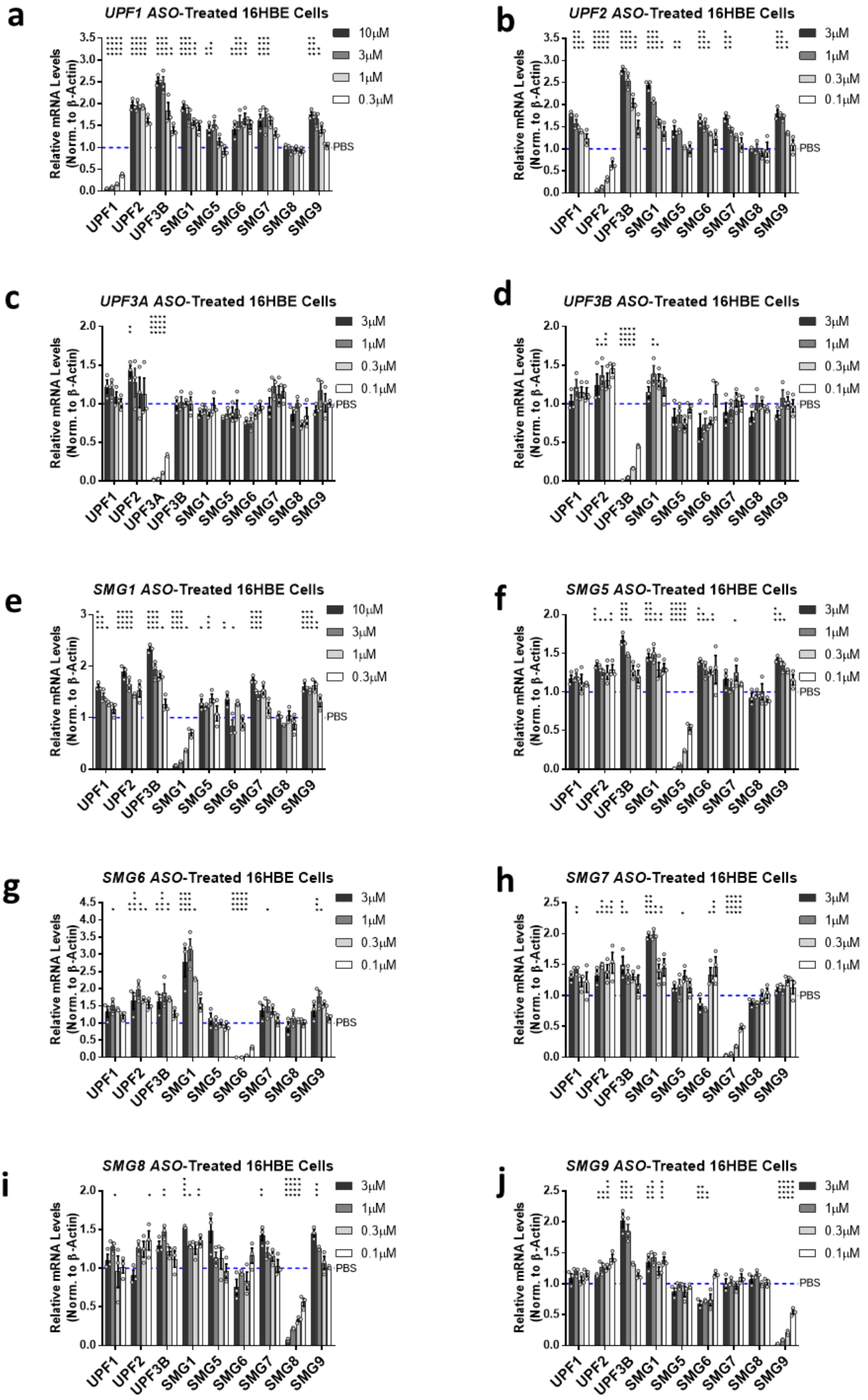

Supplementary Figure 2: Treatment of 16HBE parental cells with ASOs targeting NMD pathway components resulted in efficient target reduction and NMD pathway inhibition. RT-qPCR analysis of NMD factor mRNA levels in parental cells treated with ASO targeting (a) *UPF1*, (b) *UPF2*, (c) *UPF3A*, (d) *UPF3B*, (e) *SMG1*, (f) *SMG5*, (g) *SMG6*, (h) *SMG7*, (i) *SMG8*, and (j) *SMG9*. NMD factor mRNA levels were normalized to housekeeping gene *β-actin* mRNA levels. PBS control levels were set to 1 and are indicated by dotted blue lines. n=3 in each group. Data are presented as mean ± SEM from biologically independent samples. Statistical significance was analyzed by two-way ANOVA followed by Dunnett's multiple-comparison test (* $p < 0.05$, ** $p < 0.01$, *** $p < 0.001$, **** $p < 0.0001$). See Source Data file for the exact p values. Source data are provided as a Source Data file.

Supplementary Figure 3

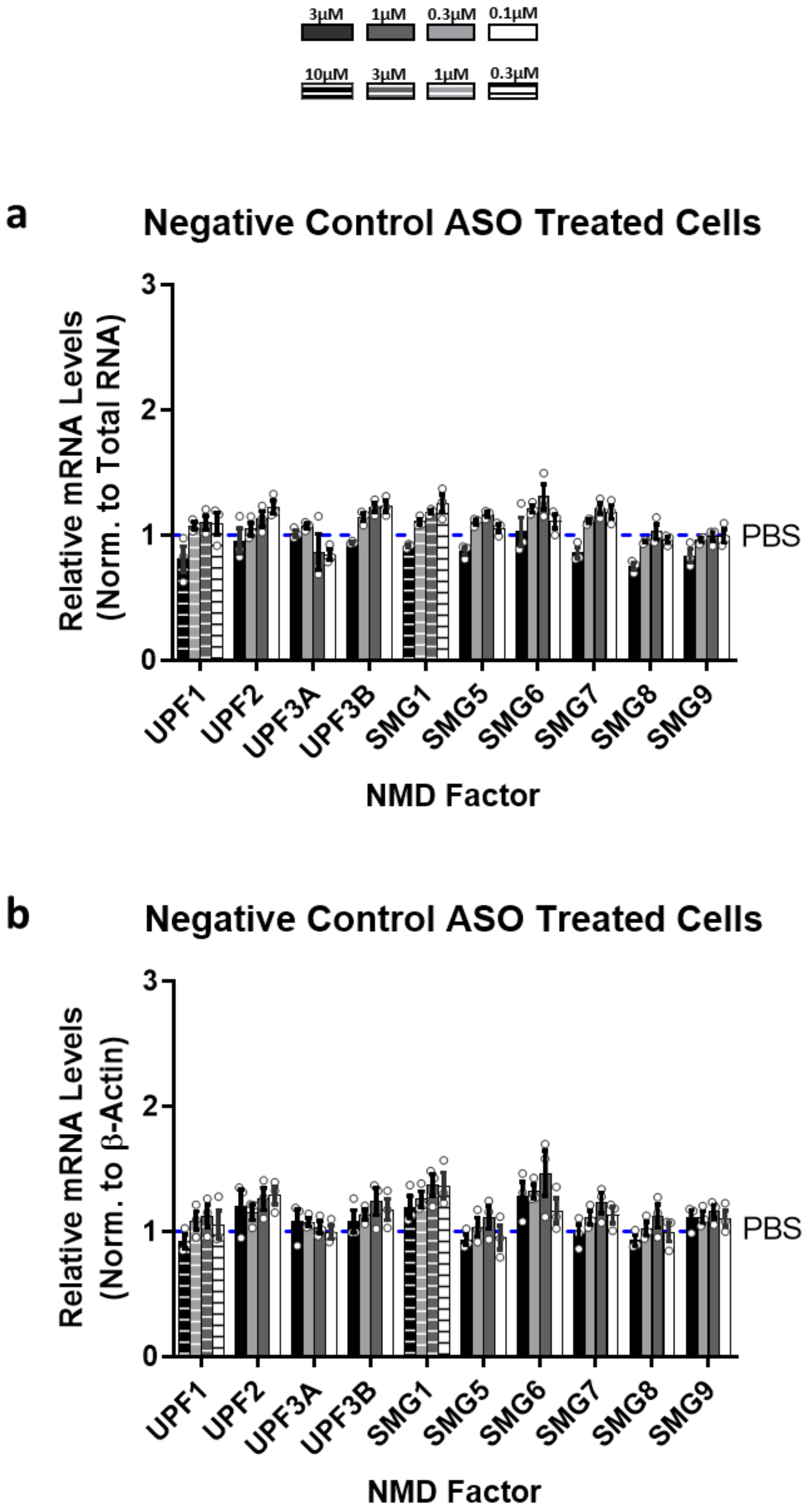

Supplementary Figure 3: Treatment of 16HBE parental cells with negative control ASO does not significantly affect NMD factor mRNA levels. RT-qPCR analysis of NMD factor mRNA levels of *UPF1*, *UPF2*, *UPF3A*, *UPF3B*, *SMG1*, *SMG5*, *SMG6*, *SMG7*, *SMG8*, and *SMG9* following treatment with negative control ASO in parental cells. NMD factor mRNA levels were normalized to (a) total RNA levels or (b) housekeeping gene *β-actin* mRNA levels. PBS control levels were set to 1 and are indicated by the dotted blue lines. n=3 in each group. Data are presented as mean ± SEM from biologically independent samples. Statistical significance was analyzed by two-way ANOVA followed by Dunnett's multiple-comparison test. See Source Data file for the exact p values. Source data are provided as a Source Data file.

Supplementary Figure 4

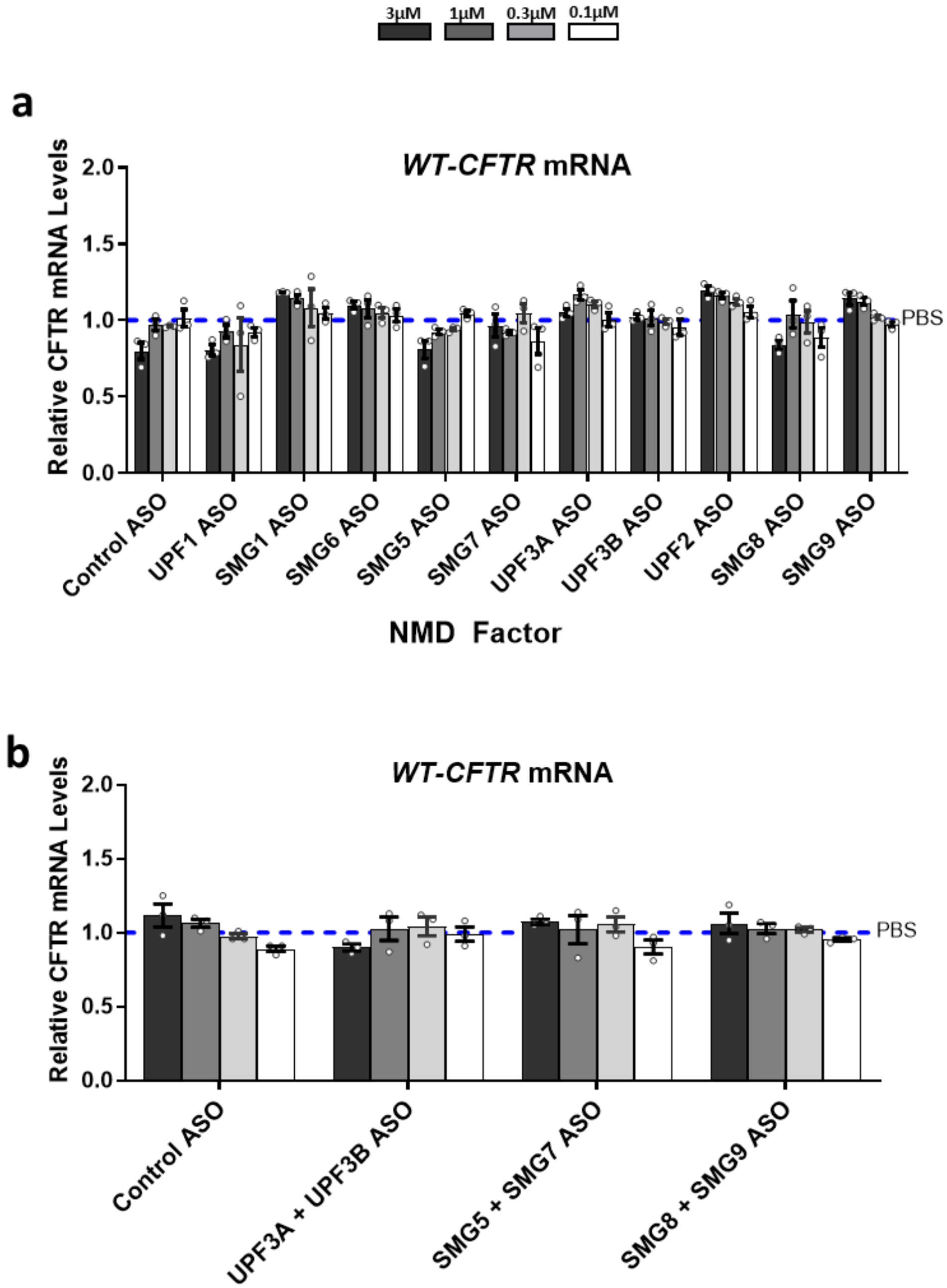

Supplementary Figure 4: ASOs targeting NMD factors do not significantly affect wild-type (WT) *CFTR* mRNA levels in 16HBE parental cells. RT-qPCR analysis of *CFTR* mRNA in parental cells treated with ASOs targeting NMD factors (a) alone or (b) in combination. *CFTR* mRNA levels in the PBS control group were set to 1 as indicated by dotted blue lines. n=3 in each group. Data are presented as mean ± SEM from biologically independent samples. Statistical significance was analyzed by two-way ANOVA followed by Dunnett's multiple-comparison test. See Source Data file for the exact p values. Source data are provided as a Source Data file.

Supplementary Figure 5

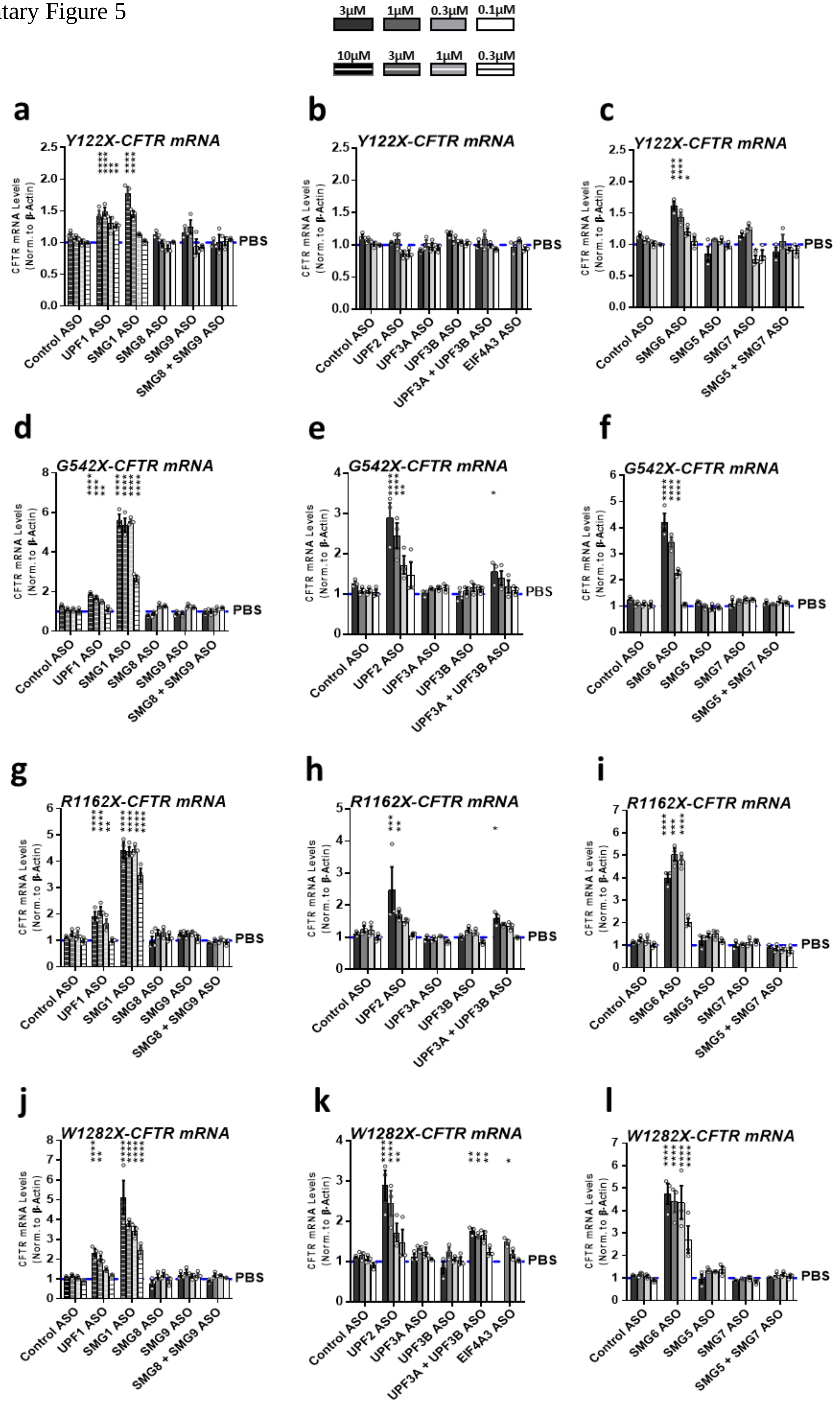

Supplementary Figure 5: *CFTR* mRNAs containing G542X, R1162X, and W1282X nonsense codons are regulated by UPF1, SMG1, SMG6, and UPF3 paralogs whereas *CFTR* mRNAs with Y122X nonsense codons are regulated by UPF1, SMG1, and SMG6 NMD factors. RT-qPCR analysis of *CFTR* mRNAs in CFF-16HBEge cells with (a-c) *CFTR-Y122X*, (d-f) *CFTR-G542X*, (g-i) *CFTR-R1162X*, and (j-l) *CFTR-W1282X* nonsense mutations with indicated ASOs targeting *UPF1, SMG1, SMG8, SMG9, UPF2, UPF3A, UPF3B, SMG6, SMG5, SMG7,* or *eIF4A3*. *CFTR* mRNA levels were normalized to *β-actin* housekeeping gene and the PBS control group was set to 1 as indicated by dotted blue lines. n=3 in each group. Data are presented as mean ± SEM from biologically independent samples. Statistical significance was analyzed by two-way ANOVA followed by Dunnett's multiple-comparison test (* $p < 0.05$, ** $p < 0.01$, *** $p < 0.001$, **** $p < 0.0001$). See Source Data file for the exact p values. Source data are provided as a Source Data file.

Supplementary Figure 6

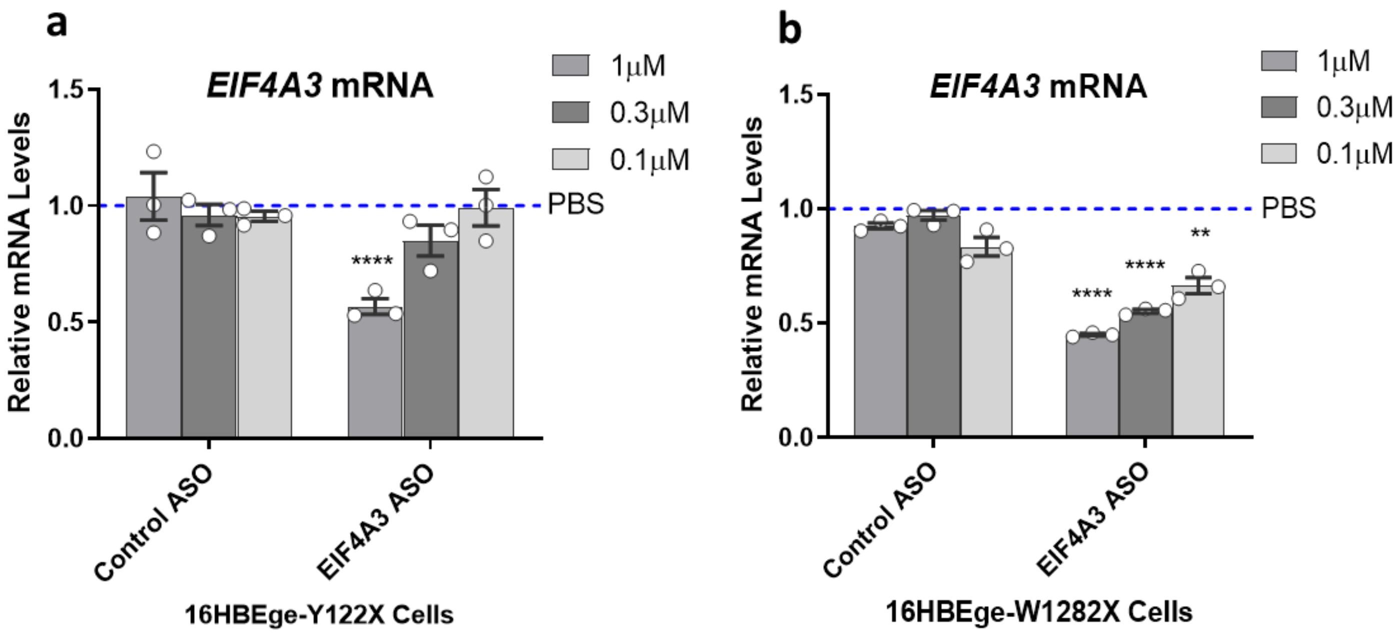

Supplementary Figure 6: *CFTR* mRNAs with W1282X but not Y122X nonsense codons are regulated by EJC core component eIF4A3. RT-qPCR analysis of *eIF4A3* mRNA in CFF-16HBEge cells harboring (a) *CFTR-Y122X* and (b) *CFTR-W1282X* CFTR nonsense mutations, following the treatment of *eIF4A3*-ASO. *eIF4A3* mRNA levels were normalized to total RNA and the PBS control group was set to 1 as indicated by dotted blue lines. n=3 in each group. Data are presented as mean ± SEM from biologically independent samples. Statistical significance was analyzed by two-way ANOVA followed by Dunnett's multiple-comparison test (** $p < 0.01$, **** $p < 0.0001$). See Source Data file for the exact p values. Source data are provided as a Source Data file.

Supplementary Figure 7

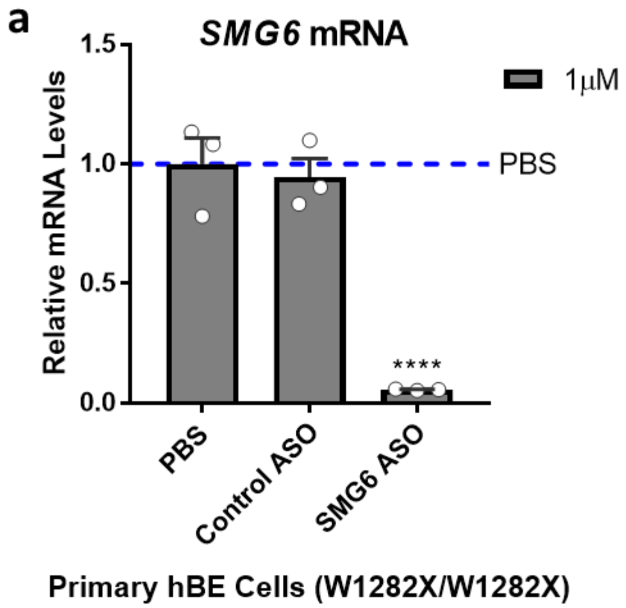

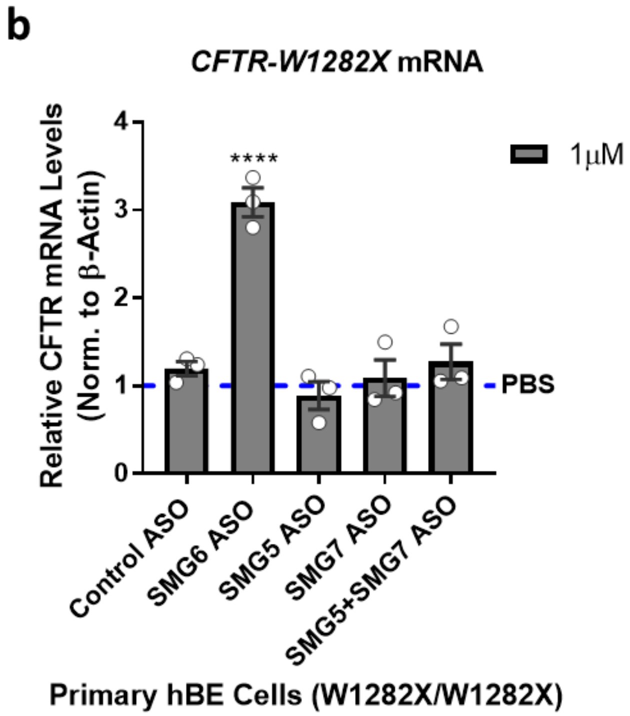

Supplementary Figure 7: *SMG6*-ASO treatment significantly upregulates *CFTR* mRNA levels in primary hBE cells harboring the homozygous *CFTR*-W1282X nonsense mutation. RT-qPCR analysis of (a) *SMG6* mRNA normalized to total RNA levels, (b) *CFTR* mRNA levels normalized to housekeeping gene *β-actin* mRNA levels, following treatment *SMG6*-ASO. PBS control groups were set to 1 as indicated by dotted blue lines. *n*=3 in each group. Data are presented as mean ± SEM from biologically independent samples. Statistical significance was analyzed by one-way ANOVA followed by Dunnett's multiple-comparison test (**** $p < 0.0001$). See Source Data file for the exact *p* values. Source data are provided as a Source Data file.

Supplementary Figure 8

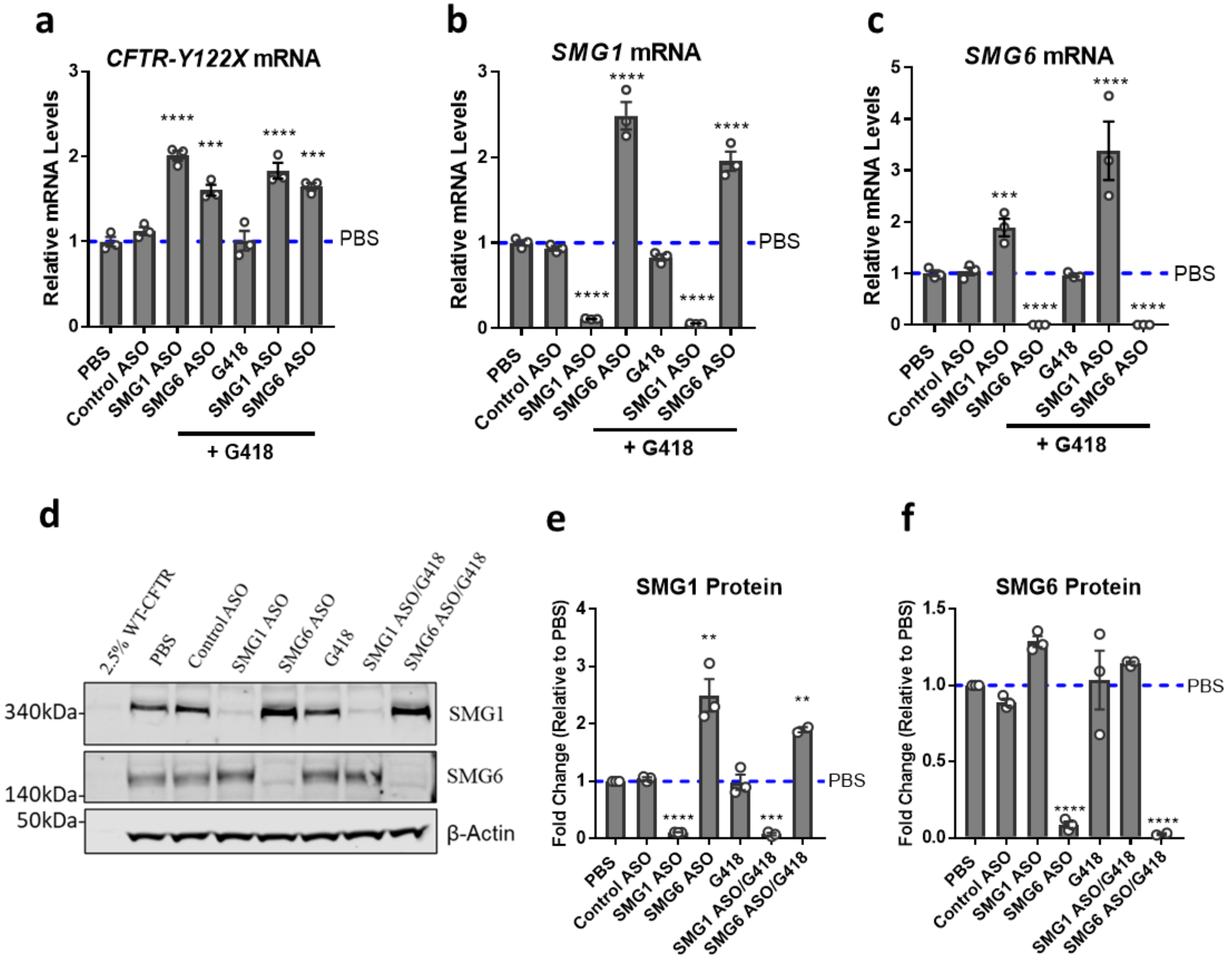

Supplementary Figure 8: *SMG1*- and *SMG6*-ASO treatments significantly upregulate CFTR-Y122X expression. CFF-16HBEge-Y122X cells were treated with *SMG1*- or *SMG6*-ASO for 6 days followed by the addition of aminoglycoside geneticin (G418; 100 µM) during the final 2 days of ASO treatments. RT-qPCR analyses of (a) *CFTR-Y122X* mRNA, (b) *SMG1* mRNA, and (c) *SMG6* mRNA following indicated treatments. Expression of mRNAs from PBS control groups was set to 1 and is indicated by dotted blue lines. (d) Qualitative western blot image and (e) quantification of SMG1 and SMG6 proteins in CFF-16HBEge-Y122X cells following indicated treatment conditions. $n=3$ in each group. Data are presented as mean ± SEM from biologically independent samples. Statistical significance was analyzed by one-way ANOVA followed by Dunnett's multiple-comparison test (** $p < 0.01$, *** $p < 0.001$, **** $p < 0.0001$). See Source Data file for the exact p values. Source data are provided as a Source Data file.

Supplementary Figure 9

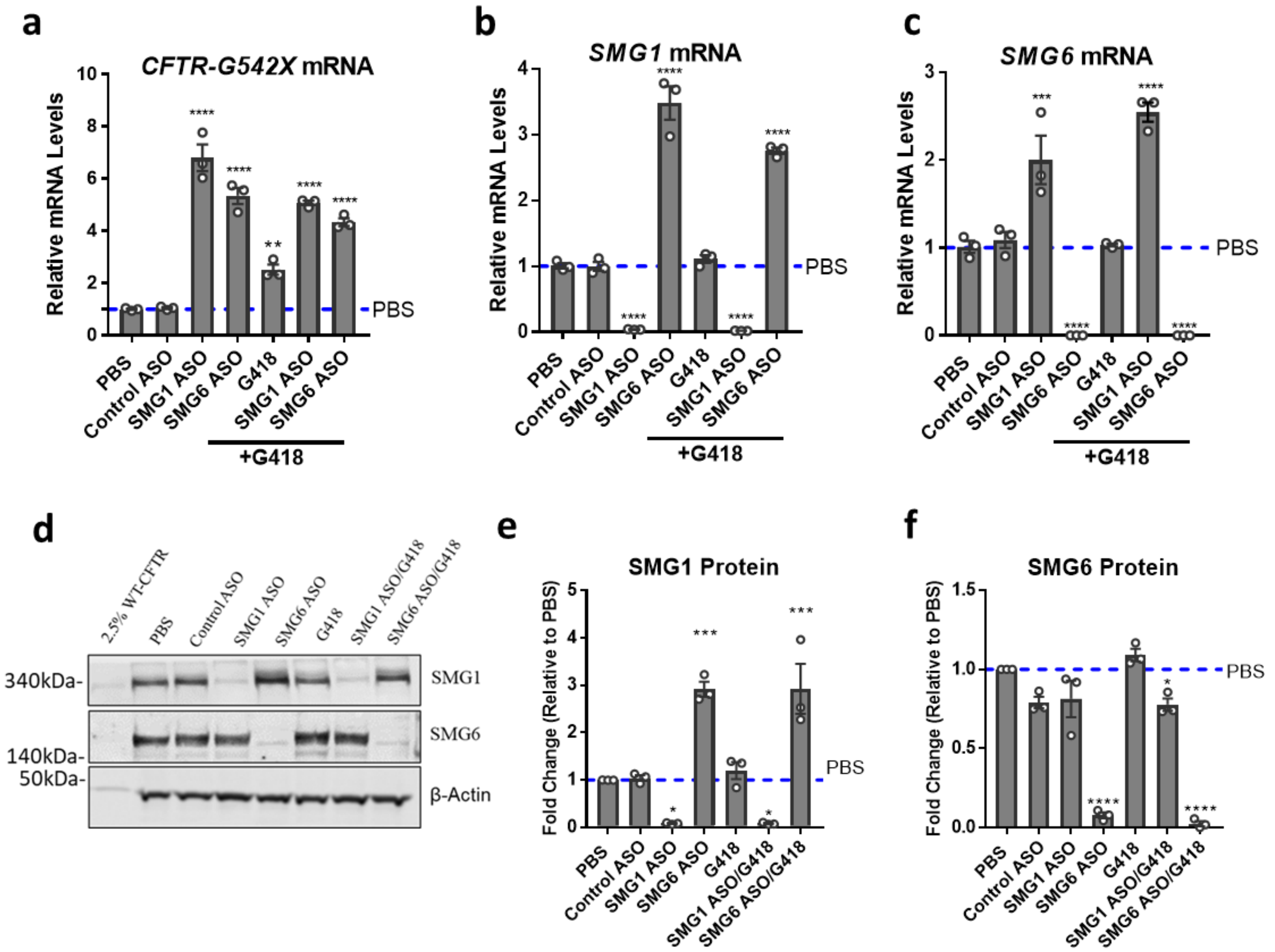

Supplementary Figure 9: *SMG1*- and *SMG6*-ASO treatments significantly upregulate CFTR-G542X expression. CFF-16HBEge-G542X cells were treated with *SMG1*- or *SMG6*-ASO for 6 days followed by the addition of aminoglycoside geneticin (G418; 100 µM) during the final 2 days of ASO treatments. RT-qPCR analyses of (a) *CFTR-G542X* mRNA, (b) *SMG1* mRNA, and (c) *SMG6* mRNA following indicated treatments. Expression of mRNAs from PBS control groups was set to 1 and is indicated by dotted blue lines. (d) Qualitative western blot image and (e) quantification of SMG1 and SMG6 proteins in CFF-16HBEge-G542X cells following indicated treatment conditions. n=3 in each group. Data are presented as mean ± SEM from biologically independent samples. Statistical significance was analyzed by one-way ANOVA followed by Dunnett's multiple-comparison test (* $p < 0.05$, ** $p < 0.01$, *** $p < 0.001$, **** $p < 0.0001$). See Source Data file for the exact p values. Source data are provided as a Source Data file.

Supplementary Figure 10

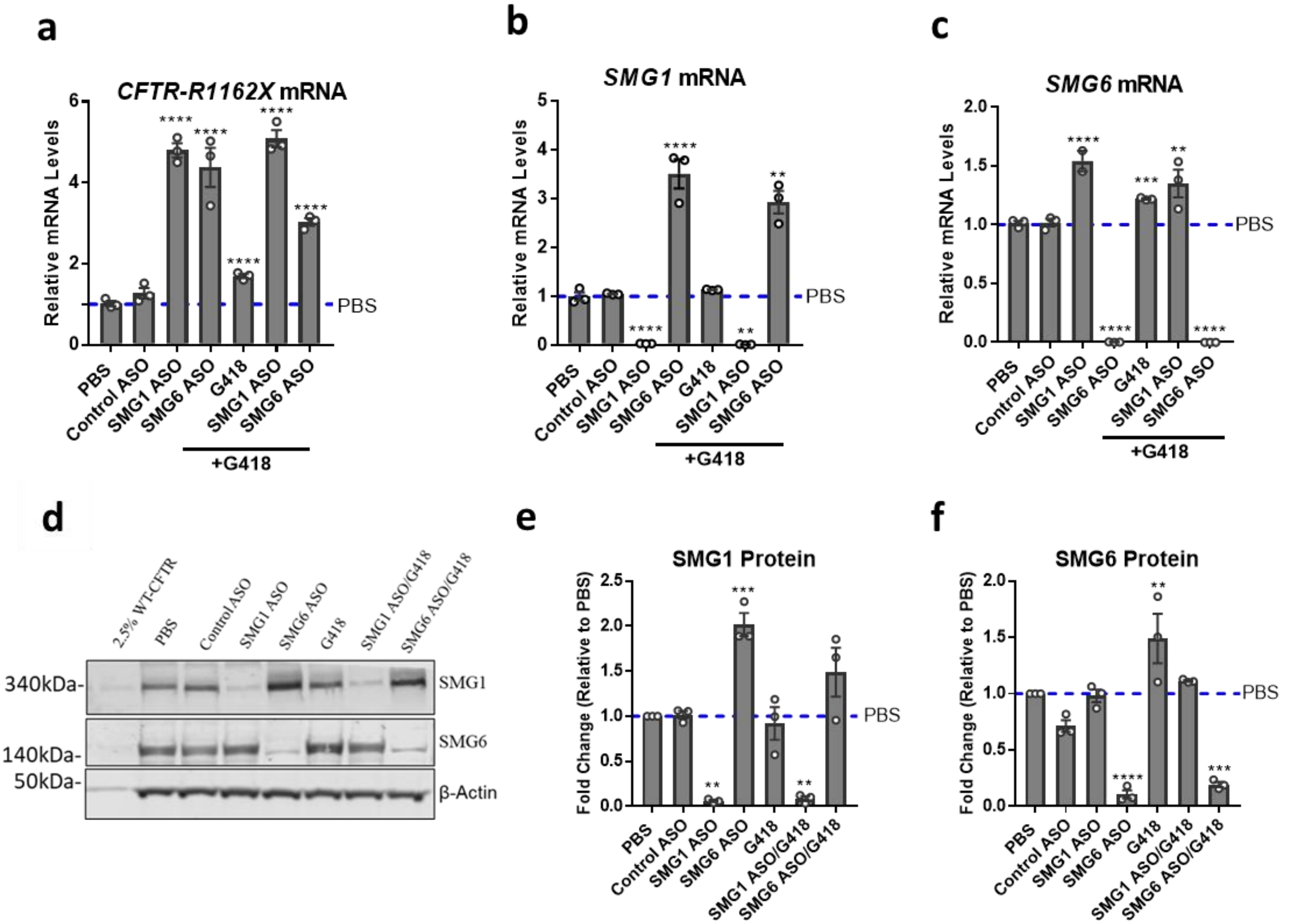

Supplementary Figure 10: *SMG1*- and *SMG6*-ASO treatments significantly upregulate CFTR-R1162X expression. CFF-16HBEge-R1162X cells were treated with *SMG1*- or *SMG6*-ASO for 6 days followed by the addition of aminoglycoside geneticin (G418; 100 µM) during the final 2 days of ASO treatments. RT-qPCR analyses of (a) *CFTR-R1162X* mRNA, (b) *SMG1* mRNA, and (c) *SMG6* mRNA following indicated treatments. Expression of mRNAs from PBS control groups was set to 1 and is indicated by dotted blue lines. (d) Qualitative western blot image and (e) quantification of SMG1 and SMG6 proteins in CFF-16HBEge-R1162X cells following indicated treatment conditions. n=3 in each group. Data are presented as mean ± SEM from biologically independent samples. Statistical significance was analyzed by one-way ANOVA followed by Dunnett's multiple-comparison test (** $p < 0.01$, *** $p < 0.001$, **** $p < 0.0001$). See Source Data file for the exact p values. Source data are provided as a Source Data file.

Supplementary Figure 11

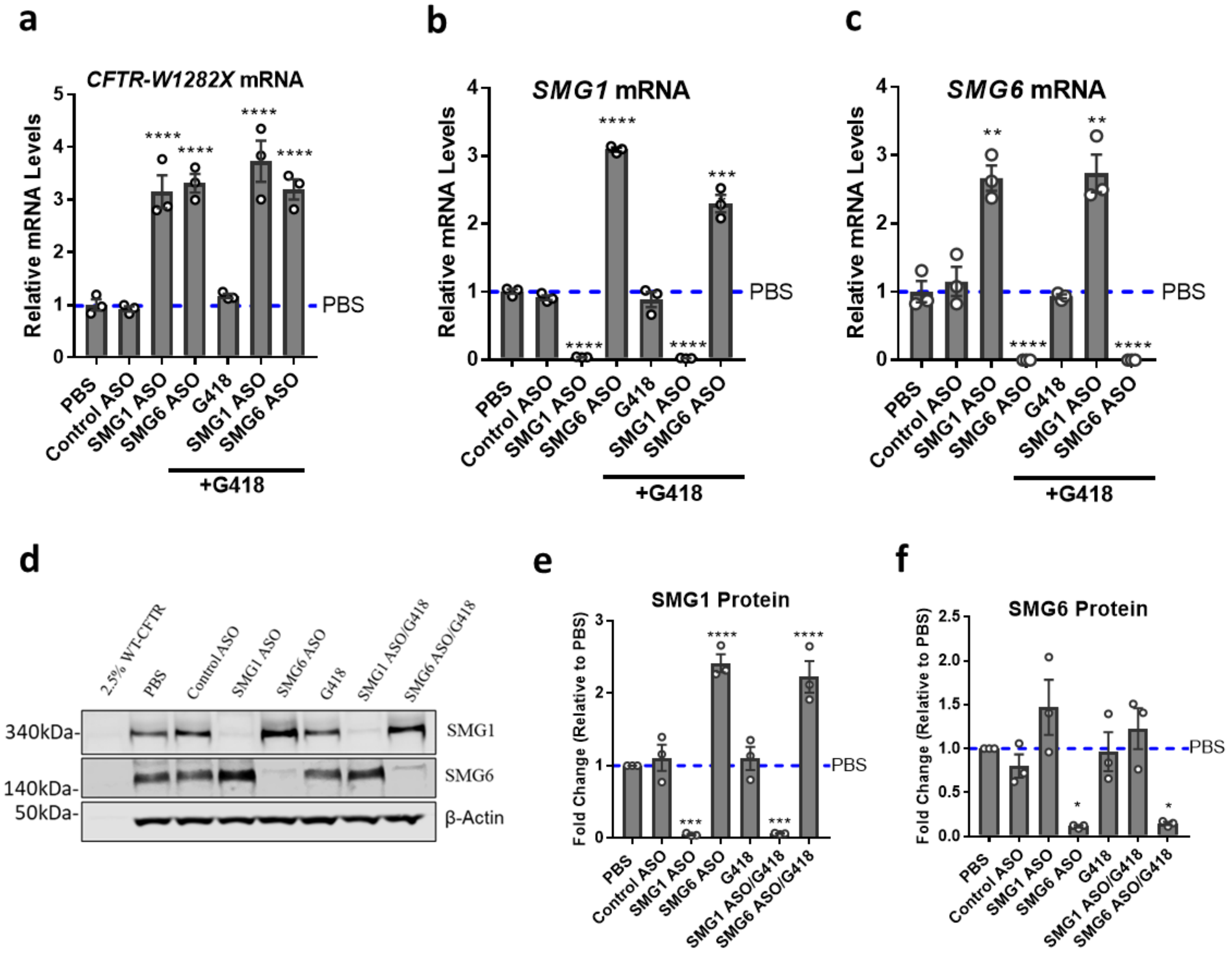

Supplementary Figure 11: *SMG1*- and *SMG6*-ASO treatments significantly upregulate CFTR-W1282X expression. CFF-16HBEge-W1282X cells were treated with *SMG1*- or *SMG6*-ASO for 6 days followed by the addition of aminoglycoside geneticin (G418; 100 µM) during the final 2 days of ASO treatments. RT-qPCR analyses of (a) *CFTR-W1282X* mRNA, (b) *SMG1* mRNA, and (c) *SMG6* mRNA following indicated treatments. Expression of mRNAs from PBS control groups was set to 1 and is indicated by dotted blue lines. (d) Qualitative western blot image and (e) quantification of SMG1 and SMG6 proteins in CFF-16HBEge-W1282X cells following indicated treatment conditions. n=3 in each group. Data are presented as mean ± SEM from biologically independent samples. Statistical significance was analyzed by one-way ANOVA followed by Dunnett's multiple-comparison test (* $p < 0.05$, ** $p < 0.01$, *** $p < 0.001$, **** $p < 0.0001$). See Source Data file for the exact p values. Source data are provided as a Source Data file.

Supplementary Table 1: Primer-probe sequences for RT-qPCR

TaqMan Primer-Probe Set	Sequence
**Human SMG1**	
Forward	AGTTCAGAATCTACTTCCAGACAC
Reverse	TCTTCGTTGTCTTCACTCTCAG
Probe	CCGACACACAGCCTGTCCAAGAAT
**Human SMG5**	
Forward	CCTCAGATTGGAATGCCCTT
Reverse	CTTGTCATACAGCCGCTTGA
Probe	TCCCATAGGCTCCCTCAAAGGACA
**Human SMG6**	
Forward	ACAAGGCTAAGGACTTCATGC
Reverse	CATTCCTTGTGAGCGCCTT
Probe	AGCACCACCTCCCGCAGTAG
**Human SMG7**	
Forward	ATCACTTCCTGCCAGTTCAG
Reverse	ATGGTTGTGTGTTGGAGGAG
Probe	TGCTTGGGTTAGAGGAATGAGGAGACT
**human SMG8**	
Forward	CCTGATGCTCCTTTGCAGAT
Reverse	GCATAAGGAAATCTCAAAACCCA
Probe	CCTGGCTGAACCTGAGGCATTAGTA
**human SMG9**	
Forward	CTGTCACACACGATCCTCAC
Reverse	TGCATGACATTCCTCTCCTTG
Probe	AAGAACTGGTTCCACTACGCTGCC
**Human UPF1**	
Forward	AGAGAGAGAGCTGCTGATGA
Reverse	CTGGGTGCTTTCGTCGATTA
Probe	CCAAGATGCAGTTCCGCTCCATTT
**human UPF2**	
Forward	AGAGTACTTTACGTCTTTGACCA
Reverse	CTGTTTATGTCTATCTTCACTGAGC
Probe	AGAATGCGCCTGTTTTGTCTCTCAGT
**human UPF3A**	
Forward	CAGTGCCAACCCTGAGAC
Reverse	TCTCTTCTCGAATTCTCTGCTTT
Probe	AGCTCATTGCTAGAAGAACCACACCTC
**human UPF3B**	
Forward	CTACTCCAGAGACACTGCTAGA
Reverse	TTCTTCTCTCATTCTCTGCTTGT
Probe	AGACAACCCCACTTTTGAGCTTCCT
**Human eIF4A3**	
Forward	GCCAACTTCACTGTATCCTCA
Reverse	AGACATCTGTAGAAATAAGCACTCG
Probe	CCCGACCGGAACTCCTTCATGAT
**Human β-Actin**	
Forward	CGGACTATGACTTAGTTGCGTTACA
Reverse	GCCATGCCAATCTCATCTTGT
Probe	CCTTTCTTGACAAAACCTAACTTGCGCAGA
**Human CFTR**	
Forward	GGCACGAAGGAGGCAGTCT
Reverse	TCGTGTGGATGCTGTTGTCTTT
Probe	ATGACACACTCAGTTAACCAAGGTCAGAACATTCAC
