# Supplementary material for: CFTR mRNAs with nonsense codons are degraded by the SMG6-mediated endonucleolytic decay pathway
Source: Nat Commun. 2022 Apr 29;13:2344. doi: 10.1038/s41467-022-29935-9 (PMC9054838; doi:10.1038/s41467-022-29935-9)
Supplement: Supplementary file 3 — Reporting Summary [file 41467_2022_29935_MOESM3_ESM.pdf]

nature portfolio

Corresponding author(s): Lulu Huang, Ph.D.

Last updated by author(s): Mar 2, 2022

Reporting Summary

Nature Portfolio wishes to improve the reproducibility of the work that we publish. This form provides structure for consistency and transparency in reporting. For further information on Nature Portfolio policies, see our Editorial Policies and the Editorial Policy Checklist.

Statistics

For all statistical analyses, confirm that the following items are present in the figure legend, table legend, main text, or Methods section.

n/a	Confirmed	
☐	☒	The exact sample size (*n*) for each experimental group/condition, given as a discrete number and unit of measurement
☐	☒	A statement on whether measurements were taken from distinct samples or whether the same sample was measured repeatedly
☐	☒	The statistical test(s) used AND whether they are one- or two-sided<br>*Only common tests should be described solely by name; describe more complex techniques in the Methods section.*
☒	☐	A description of all covariates tested
☐	☒	A description of any assumptions or corrections, such as tests of normality and adjustment for multiple comparisons
☐	☒	A full description of the statistical parameters including central tendency (e.g. means) or other basic estimates (e.g. regression coefficient) AND variation (e.g. standard deviation) or associated estimates of uncertainty (e.g. confidence intervals)
☐	☒	For null hypothesis testing, the test statistic (e.g. *F*, *t*, *r*) with confidence intervals, effect sizes, degrees of freedom and *P* value noted<br>*Give P values as exact values whenever suitable.*
☒	☐	For Bayesian analysis, information on the choice of priors and Markov chain Monte Carlo settings
☒	☐	For hierarchical and complex designs, identification of the appropriate level for tests and full reporting of outcomes
☒	☐	Estimates of effect sizes (e.g. Cohen's *d*, Pearson's *r*), indicating how they were calculated

Our web collection on statistics for biologists contains articles on many of the points above.

Software and code

Policy information about availability of computer code

Data collection	RT-qPCR: QuantStudio Real-Time PCR Software (v1.3)<br>Western blot: ImageJ (1.52a) software and Image Studio Lite (v5.2.5) software<br>Ussing Chamber: Acquire and Analyze (v2.3) software
Data analysis	GraphPad Prism version 7.01 software (GraphPad Prism, Inc., San Diego, CA) was used to perform all statistical analyses.

For manuscripts utilizing custom algorithms or software that are central to the research but not yet described in published literature, software must be made available to editors and reviewers. We strongly encourage code deposition in a community repository (e.g. GitHub). See the Nature Portfolio guidelines for submitting code & software for further information.

Data

Policy information about availability of data

All manuscripts must include a data availability statement. This statement should provide the following information, where applicable:

- Accession codes, unique identifiers, or web links for publicly available datasets
- A description of any restrictions on data availability
- For clinical datasets or third party data, please ensure that the statement adheres to our policy

All relevant data supporting the findings from this study are available within the main figures 1-7, supplementary figures 1-11, and supplementary table 1 . The raw data underlying the figures within this manuscript are included in the provided Source Data file. Antisense oligonucleotide sequences can be provided upon request. Source data are provided with this paper.

March 2021

Field-specific reporting

Please select the one below that is the best fit for your research. If you are not sure, read the appropriate sections before making your selection.

☒ Life sciences ☐ Behavioural & social sciences ☐ Ecological, evolutionary & environmental sciences

For a reference copy of the document with all sections, see nature.com/documents/nr-reporting-summary-flat.pdf

Life sciences study design

All studies must disclose on these points even when the disclosure is negative.

Sample size	No sample size calculation was performed as all independent biological replicates for all experiments were sufficient to achieve statistical power. All sample sizes are included in each figure legend.
Data exclusions	No data were excluded from analyses.
Replication	Conclusions were drawn from experiments which were successfully reproduced at least 2 times.
Randomization	Randomization was not required for in vitro experiments containing homogeneous pools of CFF-16HBEge cells. All treatments conditions were performed simultaneously with cultured cells.
Blinding	All operators performed experiments, acquired data, and performed data analysis. The experimental design employed in this study is not applicable for operator blinding.

Reporting for specific materials, systems and methods

We require information from authors about some types of materials, experimental systems and methods used in many studies. Here, indicate whether each material, system or method listed is relevant to your study. If you are not sure if a list item applies to your research, read the appropriate section before selecting a response.

Materials & experimental systems

n/a	Involved in the study	
☐	☒	Antibodies
☐	☒	Eukaryotic cell lines
☒	☐	Palaeontology and archaeology
☒	☐	Animals and other organisms
☒	☐	Human research participants
☒	☐	Clinical data
☒	☐	Dual use research of concern

Methods

n/a	Involved in the study	
☒	☐	ChIP-seq
☒	☐	Flow cytometry
☒	☐	MRI-based neuroimaging

Antibodies

Antibodies used	Mouse IgG2β anti-human CFTR (UNC596 clone; 1:2000 dilution, UNC CFTR Antibody Distribution Program, Chapel Hill, NC)<br>Mouse anti-human Na+/K+-ATPase (Santa Cruz Biotechnology, #sc-21712, 1:20000 dilution, Dallas, TX)<br>Mouse anti-human β-actin (Cell Signaling Technologies, #3700, 1:2000 dilution, Danvers, MA)<br>Rabbit anti-human SMG6 (Abcam, #ab87539, 1:2000 dilution, Cambridge, UK)<br>Rabbit anti-human SMG1 (Bethyl Laboratories, #A300-393A, 1:2000 dilution, Montgomery, TX)<br>Goat anti-mouse HRP-conjugated secondary antibody (Thermo Fisher Scientific, #31430, 1:10000 dilution, Carlsbad, CA)<br>IRDye 800CW Goat anti-Rabbit IgG (H+L) secondary antibody (Li-COR, 1:5000 dilution, #926-32211, Lincoln, NE)<br>IRDye 680RD Goat anti-Mouse IgG (H+L) secondary antibody (Li-COR, 1:5000 dilution, #926-68070, Lincoln, NE)
Validation	All antibodies used for western blotting have been validated by the manufacture and within our previous publications (Huang et.al., Genome Biology 2018 and Keenan et. al., AJRCMB 2019).

Eukaryotic cell lines

Policy information about cell lines

Cell line source(s)	CFF-16HBEge cell lines were generated and provided by the Cystic Fibrosis Foundation Therapeutics Lab (Valley et. al., JCF 2019). Primary human bronchial epithelial cells homozygous for the W1282X mutation were provided by the Cystic Fibrosis Foundation.

Authentication	No cell lines used in this study have been authenticated.
Mycoplasma contamination	Cell lines were not tested for mycoplasma contamination and no indicators of contamination were observed.
Commonly misidentified lines (See ICLAC register)	No commonly misidentified cell lines were employed in this study.
